# Current status of the treatment of microscopic polyangiitis and granulomatosis with polyangiitis in Japan

**DOI:** 10.1007/s10157-012-0651-1

**Published:** 2012-07-05

**Authors:** Koichi Sugiyama, Ken-ei Sada, Michiko Kurosawa, Jun Wada, Hirofumi Makino

**Affiliations:** 1 Department of Medicine and Clinical Science, Okayama University Graduate School of Medicine, Dentistry and Pharmaceutical Sciences, 2-5-1 Shikata-cho, Kita-ku, Okayama, 700-8558 Japan; 2 Department of Epidemiology and Environmental Health, Juntendo University School of Medicine, 2-1-1 Hongo, Bunkyo-ku, Tokyo Japan

**Keywords:** Anti-neutrophil cytoplasmic antibody (ANCA) associated vasculitis (AAV), Cyclophosphamide, Microscopic polyangiitis (MPA), Plasma exchange, Granulomatosis with polyangiitis (GPA)

## Abstract

**Background:**

This study aimed to describe the epidemiologic characteristics of microscopic polyangiitis (MPA) and granulomatosis with polyangiitis (GPA) in Japan.

**Methods:**

We used the database of the Ministry of Health, Labour and Welfare (MHLW) from 2006 to 2008, and analyzed data from 938 patients (MPA = 697, GPA = 241) who fulfilled the MHLW diagnostic criteria and had registered within a year after onset.

**Results:**

The mean ages of the MPA and GPA patients were 69.4 ± 0.4 and 58.4 ± 1.1 years, respectively. Renal (86.9 %), chest (73.7 %), and nervous system (45.2 %) symptoms were common in MPA patients. Ear, nose, and throat (86.7 %), chest (78.0 %), and renal (60.6 %) symptoms were frequently observed in GPA patients. The concomitant use of cyclophosphamide (CY) with corticosteroids was observed in 22.2 % of the MPA patients and 58.5 % of the GPA patients. In multivariate analysis, the concomitant use of CY was associated with a younger age and pulmonary hemorrhage in MPA patients, and the avoidance of CY was associated with nervous system symptoms and rapidly progressive glomerulonephritis in GPA patients. Plasma exchanges were inducted in 5.2 % of the MPA patients and 4.1 % of the GPA patients. The addition of plasma exchange was associated with elevation of the serum creatinine level in patients with both MPA and GPA.

**Conclusion:**

A dominance of MPA and a reduced frequency of renal involvement in GPA patients may be significant features of the Japanese population. Clinical practice relating to MPA and GPA in Japan can be characterized as follows: CY is used less commonly, and plasma exchange is employed for patients with deteriorated renal function.

## Introduction

Microscopic polyangiitis (MPA) and granulomatosis with polyangiitis (GPA) are the most common forms of anti-neutrophil cytoplasmic antibody (ANCA) associated diseases characterized by necrotizing small-vessel vasculitis (AAV). Approximately 90 % of patients with active, generalized MPA and GPA have circulating ANCA, and such a close association raises the possibility of pathogenic roles for ANCA. The primary antigenic targets of ANCA are myeloperoxidase (MPO-ANCA) and proteinase-3 (PR3-ANCA) of granulocytes and monocytes [1]. MPA is characterized by the frequent association of MPO-ANCA and pauci-immune necrotizing vasculitis without granulomatous lesions, while GPA is characterized by a high rate of positivity for PR3-ANCA and granuloma formation in various affected organs [2].

A previous report showed that the prevalence of MPA is higher than that of GPA in Japanese patients with renal vasculitis, while GPA is much more common in Europe [3]. It has also been reported that older Japanese patients are more likely to be affected than European patients, and that there are significant differences in the profile of ANCA positivity. Although this report demonstrated the features of Japanese patients with renal vasculitis, the characteristics and status of Japanese patients with systemic vasculitis have been not clarified. In a recommendation from EULAR, a combination therapy including cyclophosphamide (CY) and corticosteroids for inducing remission of systemic vasculitis and the need to reduce the dose according to renal function and age were proposed [4]. However, it is not known how often CY is used and what the major determinant is for the usage of CY in Japanese AAV patients in clinical practice.

In Japan, MPA and GPA have been recognized and officially certified as “intractable diseases” by the Ministry of Health, Labour and Welfare (MHLW). The patients who have MPA and GPA and wish to receive public financial aid from the MHLW to cover their medical costs must sign agreements and submit applications. Patients certified according to the MHLW diagnostic criteria for MPA or GPA [5–7] are registered in the MHLW database and information about them (clinical characteristics, medical history, organ involvements, laboratory data, and treatment modalities, are stored digitally by prefectural administrations and the MHLW.

We performed this study to survey the characteristics of Japanese patients with MPA and GPA based on a data set from the MHLW. We also attempted to investigate the frequency of the concomitant use of CY or plasma exchange with corticosteroids, and determine the factors that contributed to the choice of therapies for MPA and GPA.

## Patients and methods

### Studied patients

We were permitted to use data on 1320 patients (MPA = 988, GPA = 332) who were registered in the MHLW database from 2006 to 2008 and whose clinical data input was completed electronically. Among those 1320 patients, we identified 1032 (MPA = 787, GPA = 246) who had registered within a year after onset. We eventually used the data for the 938 (MPA = 697, GPA = 241) patients who fulfilled the MHLW diagnostic criteria. The criteria for MPA indicate the following three symptoms: rapidly progressive glomerulonephritis (RPGN), pulmonary hemorrhage, and other symptoms, including purpura, subcutaneous hemorrhage, gastrointestinal bleeding, and mononeuritis multiplex. Patients were certified as definite MPA cases if (1) they were positive for two or more of the symptoms, and had positive histological findings, or (2) they were positive for two or more of the symptoms, including RPGN and pulmonary hemorrhage, and positive for MPO-ANCA. Probable MPA cases were (1) positive for three of the symptoms or (2) positive for one of the symptoms as well as MPO-ANCA [6]. Among the 697 MPA patients, 294 were diagnosed as being definite cases and 403 were possible cases. The criteria for GPA included nose and throat (E), lung (L), and kidney (K) symptoms, as well as others due to vasculitis. Definite GPA cases were (1) positive for three or more of the symptoms, including E, L, and K symptoms, (2) positive for two or more of the symptoms and had positive histological findings, or (3) positive for one or more of the symptoms, had positive histological findings, and were positive for PR3-ANCA/C-ANCA [7]. Among the 241 GPA patients, 168 were diagnosed as definite cases and 73 were possible cases.

### Data collection and arrangement

The following information was extracted from database: sex, age, presence of a histological examination, histological findings, organ symptoms, ANCA positivity, levels of serum creatinine (Cr) and C-reactive protein (CRP), corticosteroid dosage, and concomitant usage of steroid pulse therapy, CY, and plasma exchange or hemodialysis. We categorized organ symptoms into nine groups according to the BVAS scoring system [8]: “systemic symptoms,” “cutaneous symptoms,” “mucous membranes and eye symptoms,” “ear, nose, and throat symptoms,” “chest symptoms,” “cardiovascular symptoms,” “abdominal symptoms,” “renal symptoms,” and “nervous system symptoms.” We sorted each item in the database into these categories. For example, we defined the existence of systemic symptoms as at least one “yes” among the following items: fever of 38 °C or higher for two weeks or longer, body weight loss of 6 kg or more for six months, myalgia/myositis, and arthralgia/arthritis.

### Statistical analysis

After descriptive analysis of the characteristics and treatment statuses of the MPA and GPA patients, we compared the characteristics of the patients who concomitantly use CY and those who used corticosteroid alone. We calculated the estimated glomerular filtration rate (eGFR) using the modification of diet in renal disease (MDRD) equation: 194.9 × serum Cr^−1.094.9^ × age^−0.287^ (× 0.739 if female) [9]. We categorized the patients into five groups according to eGFR and evaluated the correlation of these groups with CY usage for MPA and GPA by ANOVA. Similarly, in each disease category, we separated the patients into subgroups who did undergo plasma exchange and those who did not, and compared their characteristics. In order to identify the independent factors associated with concomitant usage of CY, the variables extracted in the univariate analysis were entered into a multivariate analysis using a logistic regression model. All of the statistical analyses in this study were performed using the statistical package of the program JMP for Windows, version 8.0 (SAS Institute Inc., Cary, NC, USA). Clinical variables that may have been related to the outcomes were compared by Mann–Whitney *U* and chi-square tests (univariate model). All results were expressed as the mean ± SE (standard error), and statistical significance was defined as a *p* value of less than 0.05 (two-tailed).

## Results

### Patient characteristics of MPA and GPA

We identified 697 MPA patients and 241 GPA patients. Patient characteristics are shown in Table 1. The mean age of the MPA patients was 69.4 ± 0.4 years, and that of the GPA patients was 58.4 ± 1.1 years. Renal involvement was most frequently observed in MPA patients (86.9 %), while ear, nose, and throat (ENT) symptoms were most common in GPA patients (86.7 %). Other major symptoms were systemic (80.3 %), chest (73.7 %), and nervous system (45.2 %) involvement in MPA patients, while systemic (81.3 %), chest (78.0 %), and renal (60.6 %) involvement were frequently noted in GPA patients. MPO-ANCA or p-ANCA was positive in 97.1 % of the MPA patients and PR3-ANCA or c-ANCA was positive in 73.0 % of the GPA patients. All but five patients with MPA and seven patients with GPA were treated with corticosteroids, and the mean maximum daily dosage of prednisolone was 26.5 ± 0.9 mg/day in the MPA patients and 35.3 ± 1.6 mg/day in the GPA patients. CY was used concomitantly by 22.2 % of the MPA patients and 58.5 % of the GPA patients. Plasma exchanges were performed in 5.2 and 4.1 % of the MPA and GPA patients, respectively.

**Table 1:** Characteristics of the MPA and GPA patients

	MPA (*n* = 697)	GPA (*n* = 241)
Diagnosis		
Definite:possible	294:403	168:73
Men:women	299:398	139:102
Age, mean ± SE years	69.4 ± 0.4	58.4 ± 1.1
Symptoms		
Systemic symptoms (%)	80.3	81.3
Cutaneous symptoms (%)	35.4	26.1
Mucous membrane and eyes (%)	13.1	46.1
Ear, nose, and throat (%)	14.1	86.7
Chest (%)	73.7	78.0
Cardiovascular (%)	14.3	15.8
Abdominal (%)	10.2	7.1
Renal (%)	86.9	60.6
Nervous system (%)	45.2	32.3
Pulmonary hemorrhage (%)	11.3	ND
Rapidly progressive glomerulonephritis (%)	63.2	25.7
Examination		
Cr, mean ± SE (mg/dl)	2.5 ± 0.1	1.6 ± 0.2
eGFR (ml/min/1.73 m^2^)	43.2 ± 1.3	75.3 ± 3.2
CRP, mean ± SE (mg/dl)	9.0 ± 0.3	10.2 ± 0.5
MPO- or p-ANCA positive (%)	97.1	17.4
PR3- or c-ANCA positive (%)	7.0	73.0
Histological finding	30.6	52
Treatment		
Max. oral PSL dosage, mean ± SE mg/day	26.5 ± 0.9	35.3 ± 1.6
m-PSL pulse (%)	51.8	38.2
Immunosuppressants usage (%)	27.8	64.3
CY usage (%)	22.2	58.5
Plasma exchange (%)	5.2	4.1
Dialysis treatment (%)	11.9	6.6

*CY* cyclophosphamide, *SE* standard error, *Cr* creatinine, *CRP* C-reactive protein, *eGFR* estimated glomerular filtration rate, *MPO-ANCA* myeloperoxidase anti-neutrophil cytoplasmic antibody, *p-ANCA* perinuclear anti-neutrophil cytoplasmic antibody, *PR3-ANCA* proteinase-3 anti-neutrophil cytoplasmic antibody, *c-ANCA* cytoplasmic anti-neutrophil cytoplasmic antibody, *PSL* prednisolone, *m-PSL* methyl prednisolone

### Characteristics of MPA and GPA patients treated with CY or corticosteroid alone

**Fig. 1:**
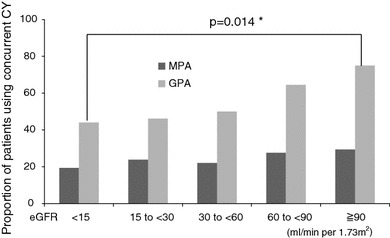
The percentage of the patients in each category of eGFR who were treated with corticosteroid plus CY. *CY* cyclophosphamide, *eGFR* estimated glomerular filtration rate, *p < 0.05

Both MPA and GPA patients treated with CY were significantly younger than those treated with corticosteroid alone (66.1 ± 0.9 vs. 70.1 ± 0.5 years, *p* = 0.0002, Table 2; and 57.2 ± 1.4 vs. 62.0 ± 1.8 years, *p* = 0.0395, Table 3, respectively). Concomitant use of CY was seen significantly less frequently in GPA patients with lower eGFR (Fig. 1, *p* = 0.014), but not significantly less frequently in such MPA patients (Fig. 1, *p* = 0.370). GPA patients with RPGN or nervous system symptoms were treated with CY less frequently than with corticosteroid alone. In contrast, MPA patients with cutaneous symptoms or pulmonary hemorrhage and GPA patients with ENT symptoms were treated with CY more frequently. The multivariate analysis included the variables extracted in the univariate analysis. Age and pulmonary hemorrhage in MPA and nervous system symptoms and RPGN in GPA were independent factors for the concomitant usage of CY (Table 4). The mean maximum daily dose of prednisone for the patients treated with corticosteroid plus CY was significant higher than the patients treated with corticosteroid alone in both MPA patients (32.6 ± 1.9 vs. 25.2 ± 1.0 mg/day, *p* = 0.0008) and GPA patients (39.4 ± 2.1 vs. 29.5 ± 2.5 mg/day, *p* = 0.0025).

**Table 2:** Characteristics of MPA patients treated with concomitant cyclophosphamide and corticosteroid monotherapy

	Concomitant CY usage (*n* = 155)	CS monotherapy (*n* = 503)	*p*
Age, mean ± SE years	66.1 ± 0.9	70.1 ± 0.5	0.0002*
Symptoms			
Systemic (%)	81.9	80.0	0.6440
Cutaneous (%)	43.2	32.0	0.0120*
Mucous membrane and eyes (%)	11.6	13.0	0.7823
Ear, nose, and throat (%)	15.5	13.4	0.5083
Chest (%)	74.8	73.0	0.6785
Cardiovascular (%)	11.0	14.8	0.2873
Abdominal (%)	12.9	9.4	0.2252
Renal (%)	85.8	87.2	0.6833
Nervous system (%)	45.8	44.2	0.7815
Pulmonary hemorrhage (%)	18.1	8.2	0.0009*
Rapidly progressive glomerulonephritis (%)	59.4	64.8	0.2518
Cr, mean ± SE mg/dl	2.1 ± 0.3	2.7 ± 0.2	0.0635
eGFR (ml/min/1.73 m^2^)	48.0 ± 2.7	41.8 ± 1.5	0.0452*
CRP, mean ± SE mg/dl	9.6 ± 0.6	8.9 ± 0.3	0.2763
Max. oral PSL dosage, mean ± SE mg/day	32.6 ± 1.9	25.2 ± 1.0	0.0008*
m-PSL pulse (%)	58.1	50.0	0.0813
Plasma exchange (%)	9.7	3.8	0.0066*
Dialysis treatment (%)	9.0	12.2	0.3146

*CY* cyclophosphamide, *CS* corticosteroids, *SE* standard error, *Cr* creatinine, *eGFR* estimated glomerular filtration rate, *CRP* C-reactive protein, *MPO-ANCA* myeloperoxidase anti-neutrophil cytoplasmic antibody, *p-ANCA* perinuclear anti-neutrophil cytoplasmic antibody, *PR3-ANCA* proteinase-3 anti-neutrophil cytoplasmic antibody, *c-ANCA* cytoplasmic anti-neutrophil cytoplasmic antibody, *PSL* prednisolone, *m-PSL* methyl prednisolone

* *p* < 0.05

**Table 3:** Characteristics of GPA patients treated with concomitant cyclophosphamide and corticosteroid monotherapy

	Concomitant CY usage (*n* = 141)	CS monotherapy (*n* = 86)	*p*
Age, mean ± SE years	57.2 ± 1.4	62.0 ± 1.8	0.0395*
Symptoms			
Systemic (%)	83.7	75.0	0.1566
Cutaneous (%)	26.2	26.3	1.000
Mucous membrane and eyes (%)	46.8	43.8	0.6760
Ear, nose, and throat (%)	90.1	80.0	0.0421*
Chest (%)	80.9	75.0	0.3106
Cardiovascular (%)	13.5	23.8	0.0637
Abdominal (%)	6.4	7.5	0.7846
Renal (%)	59.6	61.3	0.8865
Nervous system (%)	27.0	45.0	0.0077*
Rapidly progressive glomerulonephritis (%)	19.9	37.5	0.0065*
Cr, mean ± SE mg/dl	1.5 ± 0.2	1.8 ± 0.3	0.4520
eGFR (ml/min/1.73 m^2^)	80.2 ± 3.9	63.3 ± 5.3	0.0106*
CRP, mean ± SE mg/dl	10.6 ± 0.7	9.3 ± 0.9	0.2039
Max. PSL dosage, mean ± SE mg/day	39.3 ± 2.0	28.7 ± 2.7	0.0018*
m-PSL pulse (%)	39.0	41.3	0.7760
Plasma exchange (%)	5.0	2.5	0.4933
Dialysis treatment (%)	5.0	10.0	0.1711

*CY* cyclophosphamide, *CS* corticosteroids, *SE* standard error, *Cr* creatinine, *eGFR* estimated glomerular filtration rate, *CRP* C-reactive protein, *PSL* prednisolone, *m-PSL* methyl prednisolone

* *p* < 0.05

**Table 4:** Logistic regression analysis of the independent factors associated with concomitant CY use

Variable	Odds ratio (95 %CI)	*p*
MPA		
Age (per yr)	0.97 (0.96–0.99)	0.0025*
Pulmonary hemorrhage	2.76 (1.61–4.71)	0.0003*
eGFR (ml/min/1.73 m^2^)	1.00 (1.00–1.01)	0.1660
Cutanous	1.40 (0.94–2.09)	0.0986
GPA		
Age (per year)	0.99 (0.97–1.00)	0.1889
ENT symptoms	1.96 (0.85–4.60)	0.1151
Nervous system symptoms	0.48 (0.26–0.88)	0.0174*
RPGN	0.45 (0.24–0.87)	0.0165*

*ENT* ear, nose, and throat, *eGFR* estimated glomerular filtration rate, *RPGN* rapidly progressive glomerulonephritis

* p < 0.05

### Patient characteristics of MPA and GPA treated with or without plasma exchange

The mean ages of the patients who underwent plasma exchange and those who did not were similar for both the MPA (68.6 ± 1.9 vs. 69.4 ± 0.5 years, *p* = 0.7041; Table 5) and the GPA (57.9 ± 5.3 vs. 58.5 ± 1.1 years, *p* = 0.9162; Table 6) patients. The MPA patients who were treated with plasma exchange had pulmonary hemorrhages more frequently than those who were not (18.1 vs. 8.2 %, *p* = 0.0009). The serum levels of Cr in patients who underwent plasma exchange were higher than the levels in those who did not undergo plasma exchange for both the MPA (3.8 ± 0.5 vs. 2.4 ± 0.1 mg/dl, *p* = 0.0132) and the GPA (3.7 ± 0.8 vs. 1.5 ± 0.2, *p* = 0.0102) patients. Furthermore, the patients treated with plasma exchange had RPGN more frequently than those who were not treated with it among GPA patients, and all of the GPA patients who underwent plasma exchange had renal symptoms. Similarly, dialysis treatment was more frequent in the patients who underwent plasma exchange than in those who did not among MPA patients (50.0 vs. 9.8 %, *p* < 0.0001) and among GPA patients (60.0 vs. 4.3 %, *p* < 0.0001).

**Table 5:** Characteristics of MPA patients who did and did not undergo plasma exchange

	PE (*n* = 36)	Non-PE (*n* = 661)	*p*
Age, mean ± SE years	68.6 ± 1.9	69.4 ± 0.5	0.7041
Symptoms			
Systemic (%)	69.4	80.9	0.1280
Cutaneous (%)	44.4	35.0	0.2835
Mucous membrane and eyes (%)	27.8	12.3	0.0178*
Ear, nose, and throat (%)	16.7	13.9	0.6229
Chest (%)	83.3	73.2	0.2426
Cardiovascular (%)	25.0	13.8	0.0827
Abdominal (%)	8.3	10.3	1.0000
Renal (%)	97.2	86.4	0.0722
Nervous system (%)	33.3	45.8	0.1697
Pulmonary hemorrhage (%)	36.1	10.0	<0.0001*
Rapidly progressive glomerulonephritis (%)	72.2	62.8	0.2902
Cr, mean ± SE mg/dl	3.8 ± 0.5	2.4 ± 0.1	0.0132*
eGFR (ml/min/1.73 m^2^)	26.7 ± 5.7	44.1 ± 1.3	0.0031*
CRP, mean ± SE mg/dl	12.2 ± 1.1	8.8 ± 0.3	0.0037*
Max. oral PSL dosage, mean ± SE mg/day	23.5 ± 4.2	26.7. ± 0.9	0.4580
m-PSL pulse (%)	86.1	49.9	<0.0001*
CY usage (%)	41.7	21.2	0.0068*
Dialysis treatment (%)	50.0	9.8	<0.0001*

*CY* cyclophosphamide, *SE* standard error, *Cr* creatinine, *eGFR* estimated glomerular filtration rate, *CRP* C-reactive protein, *PSL* prednisolone, *m-PSL* methyl prednisolone

* p < 0.05

**Table 6:** Characteristics of GPA patients who did and did not undergo plasma exchange

	PE (*n* = 10)	Non-PE (*n* = 231)	*p*
Age, mean ± SE years	57.9 ± 5.3	58.5 ± 1.1	0.9162
Symptoms			
Systemic (%)	80.0	81.4	1.000
Cutaneous (%)	40.0	25.5	0.2931
Mucous membrane and eyes (%)	40.0	46.3	0.7566
Ear, nose, and throat (%)	80.0	87.0	0.6262
Chest (%)	90.0	77.5	0.6958
Cardiovascular (%)	40.0	14.7	0.0547
Abdominal (%)	10.0	6.9	0.5258
Renal (%)	100.0	58.9	0.0071^*^
Nervous system (%)	50.0	31.6	0.3000
Rapidly progressive glomerulonephritis (%)	60.0	24.2	0.0203^*^
Cr, mean ± SE mg/dl	3.7 ± 0.8	1.5 ± 0.2	0.0102^*^
eGFR (ml/min/1.73 m^2^)	33.1 ± 15.5	77.1 ± 3.2	0.0058^*^
CRP, mean ± SE mg/dl	11.2 ± 2.6	10.2 ± 0.5	0.7008
Max. oral PSL dosage, mean ± SD mg/day	20.0 ± 8.4	35.9 ± 1.6	0.0663
m-PSL pulse (%)	90.0	35.9	0.0009^*^
CY usage (%)	70.0	57.6	0.5268
Dialysis treatment (%)	60.0	4.3	<0.0001^*^

*PE* plasma exchange, *SE* standard error, *Cr* creatinine, *eGFR* estimated glomerular filtration rate, *CRP* C-reactive protein, *PSL* prednisolone, *m-PSL* methyl prednisolone

*p < 0.05

## Discussion

We determined the characteristics and the current treatment statuses of MPA and GPA patients based on a data set from the MHLW database in Japan. In the present study, the ratio of the patients with MPA to the patients with GPA was 3:1. Watts et al. [10] reported that the ratio of MPA patients to GPA patients was 3:4 in the UK. The predominance of MPA compared with GPA in Japan seen in our study was similar to that noted in a previous report [3, 11]. MPO-ANCA- or p-ANCA-positive MPA patients and PR3-ANCA- or c-ANCA-positive GPA patients were more common in this study. These results are consistent with a previous Japanese nationwide epidemiological survey which showed that 87.3 % of MPA patients were p-ANCA positive and 85.7 % of WG patients were c-ANCA positive [11]. A report on a Western patient population showed that 30.4 % of MPA patients were MPO-ANCA or p-ANCA positive and 57.4 % of GPA patients were PR3-ANCA or c-ANCA positive [12]. The MHLW criteria for MPA and GPA consist of three parts: clinical symptoms, histological findings, and ANCA positivity. The MPA criteria include MPO-ANCA or p-ANCA positivity while the GPA criteria include PR3-ANCA or c-ANCA positivity. There is no reference to ANCA in the classification criteria of the American College of Rheumatology (ACR) or the Chapel Hill Consensus Conference. Although Watts et al. included ANCA positivity in their classification algorithm, the type of ANCA was not taken into account in the diagnosis of MPA and GPA. These differences between the MHLW criteria and other criteria could have affected the numbers of ANCA-positive GPA and MPA patients in our study. In addition, the MHLW criteria emphasize specific clinical symptoms, such as RPGN and pulmonary hemorrhage, meaning that our study population may differ from those in previous reports. Renal involvement was noted in only 60 % of patients with GPA in the present study, even if all renal symptoms were included; glomerulonephritis occurred in almost 80 % of GPA patients during their clinical courses in the USA [2]. Only patients who had registered within a year after disease onset were included in this study. In addition, the MHLW criteria for possible GPA cases comprise fewer clinical symptoms than the ACR criteria for diagnosing GPA. Therefore, more organ-limited patients may be present in this study population.

Concomitant CY usage was a less common option for treatment of AAV in the present study. Only 22 % of the patients with MPA and 59 % of the patients with GPA were treated with CY combined with corticosteroids, despite the fact that several guidelines recommend the concomitant usage of CY for AAV [4, 13]. Indeed, 89 % of the AAV patients were treated with CY combined with corticosteroids according to a previous report on a European population [14]. Even in another Asian country, 94 % of the MPA patients were treated with a combination of CY and corticosteroids [15]. A previous nationwide survey of the Japanese population showed that CY was used by 62.3 % of MPA and 96.3 % of GPA patients [11], and a recent study of Japanese patients with MPO-ANCA-associated vasculitis (JMAAV) also reported that 58.3 % of the patients were treated with CY [16]. Therefore, less common usage of CY may be a characteristic of the current Japanese therapy for AAV. In the multivariate analysis, avoidance of concomitant CY usage was associated with older age and pulmonary hemorrhage in MPA patients, and RPGN and nervous system symptoms in GPA patients. Several guidelines recommend that the dosage of CY should be reduced for elderly patients or those with deteriorated renal function in order to avoid adverse effects [4, 13]. Additionally, a Japanese nationwide survey of RPGN reported that there was no additional benefit of immunosuppressants for the renal prognoses of elderly patients based on data for 715 RPGN patients collected until 2001 [17]. Therefore, avoidance of concomitant usage rather than proactive usage with the dose of CY reduced for elderly patients and patients with RPGN may be a significant feature of the current treatment approach for MPA in Japan.

The maximum daily dose of corticosteroids was higher in patients who concomitantly used CY than in those who did not. During the treatment of AAV patients, it is common practice for the initial dose of corticosteroids to be continued for one month, whereas a more rapidly tapering regimen of corticosteroids was adopted in several recent clinical trials (patients initially received 1 mg/kg/day of oral prednisolone, which was reduced to 0.75 mg/kg/day after one week, and 0.50 mg/kg/day after two weeks) [18–20]. Because the body weights of patients with AAV were not registered in the database, we were not able to convert the daily dose of corticosteroids per body into the dose per kilogram in this study. However, AAV patients are commonly treated with about 0.8 mg/kg/day of oral prednisolone in Japan. Although the rate of tapering of corticosteroids could not be assessed in present study, patients who are concomitantly using CY may be treated with a higher dose of corticosteroid and tapered more rapidly, as in the regimen applied in recent clinical trials.

In our study, about 5 % of the patients were treated with plasma exchange. This result is similar to that seen in the JMAAV study, which reported that plasma exchange was applied in only 2 of 48 patients [16]. A recent Western study of remission maintenance showed that almost 15 % of patients with AAV were treated with plasma exchange, and the median level of serum Cr was 2.9 or 2.7 mg/dl [18]. The addition of plasma exchange was associated with an elevation in the serum creatinine level in both MPA and GPA patients. Plasma exchange may be initiated based on renal dysfunction rather than disease classification. The European randomized Methylprednisolone versus Plasma Exchange (MEPEX) trial showed that plasma exchange increased the rate of renal recovery in ANCA-associated systemic vasculitis that presented with renal failure [20]. The EULAR recommendations propose the concomitant usage of plasma exchange for patients with rapidly progressive severe renal disease based on this clinical trial [4]. Because of the less severe deterioration in renal function observed in our population, the concomitant usage of plasma exchange may have been less common. Among all chest symptoms, plasma exchange was performed most commonly in patients with pulmonary hemorrhage in our study. The British Society for Rheumatology and British Health Professionals in Rheumatology guidelines for vasculitis recommend that treatment with plasma exchange should be considered in patients with life-threatening manifestations of disease such as pulmonary hemorrhage as well as in patients presenting with severe renal failure [13].

Several limitations of the present study should be noted. First, we were not able to reconfirm the clinical data by checking medical records. Sakauchi et al. [21], who studied the etiology of primary biliary cirrhosis using the MHLW database, noted a similar limitation. In addition, we cannot discuss the reliability of the data used in the present study since this is the first report on the characteristics of MPA and GPA patients in Japan. Secondly, we applied the MHLW criteria to diagnose MPA and GPA patients in the present study; however, the specificity and sensitivity of the MHLW criteria have not yet been validated. Third, the choice of therapeutic modality may be influenced by healthcare access. Finally, our investigation failed to demonstrate an association between these treatments and prognosis since it was a cross-sectional study.

In conclusion, the dominance of MPA patients over GPA patients and a lower frequency of renal involvement in GPA patients may be significant features of Japanese MPA and GPA patients. Concomitant CY usage was relatively less commonly used to treat AAV in Japan. Plasma exchange was used in AAV patients with deteriorated renal function. Further investigations based on global definitions are required to further confirm these features of Japan patients.
